# A Potential Role of EFR3A in Human Disease States

**DOI:** 10.3390/biom15040466

**Published:** 2025-03-22

**Authors:** Karolina Marek-Bukowiec, Magdalena Trybus, Anita Hryniewicz-Jankowska, Aleksander Czogalla, Aleksander F. Sikorski

**Affiliations:** 1 Research and Development Centre, Regional Specialist Hospital, ul. Kamieńskiego 73a, 51-124 Wroclaw, Poland; karolina.marek-bukowiec@umw.edu.pl (K.M.-B.); magdalenatrybus89@gmail.com (M.T.); 2 Department of Cytobiochemistry, Faculty of Biotechnology, University of Wroclaw, ul. Joliot-Curie 14a, 50-383 Wroclaw, Poland; anita.hryniewicz-jankowska@uwr.edu.pl

**Keywords:** EFR3, EFR3A, EFR3B, membrane protein, phosphatidylinositol-4 kinase, neuronal, cardiovascular diseases, cancer

## Abstract

EFR3A is a conserved peripheral membrane protein required for the plasma membrane localization of the phosphatidylinositol-4 kinase (PI4KIIIα/PI4KA) complex and for regulating the responsiveness of G-protein-coupled receptors. Additionally, it was implicated in several other potentially unrelated physiological functions. In metazoan organisms, *EFR3A* is ubiquitously co-expressed with its paralog *EFR3B* which shares similar biological roles. This brief review summarizes the current knowledge regarding the potential roles of EFR3A in human disease states, including neurological and cardiovascular disorders, as well as various neoplasia-based diseases.

## 1. Introduction

**Figure 1:**
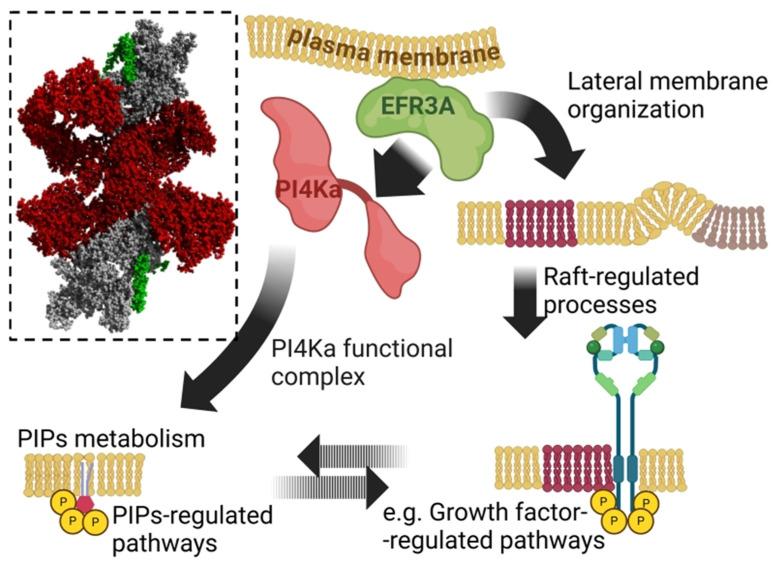
Schematic representation of experimentally proven (solid arrows) and putative (dashed arrows) functions of EFR3A. The protein is involved in the spatiotemporal organization of the phosphatidylinositol 4-kinase (PI4KIIIα/PI4KA) complex, thus influencing phosphoinositide (PIP) and G-protein signaling pathways. Inset: full atom representation of PI4KA tandem (red) bound to C-terminus of EFR3A (green) together with TTC7B and FAM126 (gray)—rendered with YASARA v.20.12.24 [14] based on PDB ID: 9BAX [3]. EFR3A also modulates the lateral organization of the plasma membrane and influences epidermal growth factor receptor (EGFR) and phospholipase C gamma phosphorylation. Scheme created with BioRender.com. For further details, see [4].

Although EFR3A was discovered over three decades ago, its structure, biological roles, and contribution to human pathologies remain largely understudied. The *EFR3A* gene (formerly known as *KIAA0143*) encodes a conserved in evolution, peripheral membrane protein best-known for its role in the spatiotemporal organization of the phosphatidylinositol 4-kinase (PI4KIIIα/PI4KA) complex at the plasma membrane (PM) (Figure 1) [1,2,3,4]. PI4KA is the key enzyme converting PI into PI4P which is a signaling molecule involved in, e.g., exocytic and autophagy pathways [5,6] and a substrate mainly for synthesis of PI(4,5)P_2_, one of most important signaling molecules involved in many pathways, among others such as Hippo [7] and Toll-like receptors signaling pathways [8,9], and autophagy [5,10]. However, the major consumer of the PI(4,5)P_2_ are trimeric protein G-coupled phospholipase C signaling pathways that regulate essential cellular processes such as cell proliferation, motility, and apoptosis [11]. PI4KA is one of four major isoforms encoded by distinct genes predominantly responsible for maintaining the PM pool of PI(4,5)P_2_ [12]. Thus, structural alterations at the molecular level resulting from mutations, changes in post-translational modifications, or deficiencies/excesses of this protein and other members of the PI4KA complex can disrupt cellular homeostasis, potentially contributing to the pathology of mammalian organisms [13]. An increasing number of studies have identified significant correlations between mutational and expressional abnormalities in *EFR3A* and various human diseases, including neurological disorders, cardiovascular conditions, and cancer [13]. Comprehending EFR3A’s roles in human health and disease may provide critical insight into the mechanisms underlying these conditions and aid in developing novel diagnostic and therapeutic strategies for future clinical applications. In this brief review, we summarize available information linking EFR3A to human disease states including autism, glioma, cardiovascular diseases, and colorectal and pancreatic cancer.

## 2. EFR3A—History in a Nutshell

The *EFR3A* gene, initially designated as *KIAA0143* (human *KIAA0143*, *hKIAA0143*), was first cloned and mapped in the human genome (macrophage KG-1 cell line) in 1995 by Nagase et al. [15]. The gene was predicted to encode an 885 amino acid protein with five putative transmembrane domains. It was demonstrated to be actively transcribed across various human tissues, including the heart, brain, placenta, lung, liver, skeletal muscle, kidney, pancreas, spleen, thymus, prostate, testis, ovary, small intestine, colon, and peripheral blood leukocytes [15,16].

In 1998, Faulkner et al. [17] cloned the *D. melanogaster* homolog of the *hKIAA0143* gene (*cmp44E*), encoding a protein also called RBO [18] that shared ~34% amino acid sequence identity with the human counterpart. The *cmp44E* mRNA was present in virtually every organ of *Drosophila* at all developmental stages. It was particularly abundant in the central nervous system (CNS) from mid-embryogenesis to the adult stage. It was shown that the *cmp44E* gene is essential for fruit fly development, as its inactivation by homozygous deletion of the entire or significant part of the coding region resulted in 100% embryonic lethality [17]. Interspecies comparison of the genomes available in the 1990s allowed the identification of sequences similar to *hKIAA0143* and *cmp44E* in distantly related species, i.e., yeast, *S. cerevisiae* (*EFR3*, 12% sequence identity), *C. elegans* (*C32D5.3*, 33% sequence identity), and in a model plant, *A. thaliana* (*A_IG002P16.24*, 15% sequence identity). These early studies indicated significant similarity at the primary structure level but also suggested the presence of different numbers (from two to five) of the presumptive transmembrane domains of distinct membrane topologies [17,18,19]. The most recent structural studies on yeast EFR3 protein along with AI-based computer models of ERF3A and its orthologs do not confirm the presence of transmembrane helices [18]; see also, e.g., [AF_AFQ8IGJ0F1] [20]. A year later, the cDNA clone from the human brain was characterized and identified as a novel *KIAA0953* gene which appeared to be a paralog of *KIAA0143* (around 60% amino acid similarity). Although the paralog mRNAs could be detected in numerous human tissues, their expression pattern varies. While *KIAA0953* was most abundant in the brain and least abundant in the kidneys and the skeletal muscles, the *KIAA0143* expression profile was the opposite [15,21]. Later, *KIAA0143* was renamed *EFR3A,* while *KIAA0953* became *EFR3B* [22]. In this review, we will focus mostly on EFR3A, which is better known. Next, the study by Munemoto et al. [23] identified the mouse homolog of the human *KIAA0143* gene (*mKIAA0143*) and linked its dysregulation in auditory brainstem neurons (at the mRNA level) to hearing deficiency in mice. The hypothetical mKIAA0143 protein, consisting of 819 amino acid residues, was found to be nearly 97% identical to human EFR3A. Subcellular tracking of the EGFP-tagged mKIAA0143 chimera in monkey COS-1 kidney fibroblasts cell line revealed that the protein is efficiently targeted to the plasma membrane [23].

Another study on *Drosophila* showed that RBO acts as a plasma membrane protein engaged in G-protein signaling mediated phototransduction [18]. The conclusion was derived from the observation of mutant flies bearing temperature-sensitive mutations in the *cmp44E* locus, which manifested immediate reversible blindness at a restrictive temperature (37 °C). The conditional loss of *cmp44E* resulted in early termination of G-protein-coupled receptor (GPCR) signaling and caused inhibition of the phospholipase Cβ (PLCβ)-dependent opening of light-sensitive channels (TRP and TRPL). Due to the characteristic temperature-sensitive phenotype of the conditional mutant, the *cmp44E* gene obtained an alternative descriptive name, “rolling blackout” (*rbo*) [18]. Further studies of temperature-sensitive mutants implied a key role for cmp44E in vesicular trafficking, particularly clathrin-independent endocytosis. The effect of Cmp44E loss was observed in both non-neuronal and neuronal cells [19]. According to the authors, the temperature-sensitive mutation also affected syntaxin-1A-dependent exocytosis which is responsible for cellular processes underlying phototransduction [24,25]. One of the aforementioned studies [18] clarified that the *cmp44E/rbo* is, in fact, the *Stambh A* (*StmA*) locus, first mapped by Shyngle and Sharma in 1995 [26], three years before Faulkner cloned *cmp44E*. Consequently, the Human Genome Nomenclature Committee (HGNC) approved *Stambh A* as the official gene name, retaining *cmp44E* and *rbo* as synonyms.

After the first in vivo map of the yeast interactome was published, insight into the interaction network of EFR3 became possible [27]. The yeast homolog was found to interact with five proteins, i.e., plasma membrane proteins Mid2, Ypp1, Agp1, the endoplasmic-reticulum-associated protein Rtn1, and the fungi-specific Ppz1 phosphatase [27]. As Mid2, Ypp1, Agp1, and Rtn1 are orthologs of human MID2, TTC7A, AGP1, and RTN1 proteins, respectively, the results pointed at possible binding partners of the human EFR3A protein.

An independent study published the same year confirmed the interaction between EFR3, Ypp1, and Stt4 kinase (PI4-kinase in humans) in vitro and evidenced its key role in the formation and stabilization of the complex at membrane PI kinase patches [28]. EFR3 turned out to be the only component of the complex possessing a membrane-binding motif. The elimination of EFR3 prevented the assembly of the membrane kinase complex, caused Stt4 mislocalization to the cytoplasm, and led to a drastic decrease in PI4P levels in the plasma membrane [28]. A wider characterization of the mammalian EFR3s (mice mKIAA0143 and human hKIAA0143) comes from the study by the De Camilli group. The study demonstrated that mammalian EFR3s are, in fact, peripheral proteins that require palmitoylation at their N-terminal cysteine-rich motifs to translocate from the cytosol to the plasma membrane (mechanism conserved in metazoan organisms but not in yeast). Similarly to the yeast EFR3, the mouse and human orthologs were found to bind PI4KA and TTC7 protein (homolog of yeast Ypp1) and assemble them into a functional complex required for constant resynthesis of PI4P at the plasma membrane [1,2].

Further studies uncovered an important role for the human EFR3 paralogs in regulating responsiveness to G-protein-coupled receptors (GPCRs), specifically the angiotensin receptor type 1 (AT1R). Bojjireddy et al. [22] showed that deleting *EFR3A* and *EFR3B* in human HEK293 cells caused the hyperphosphorylation of AT1R and its faster desensitization. These findings agreed with observations made in *Drosophila*, in which *cmp44E* (*rbo*) loss resulted in the termination of GPCR signaling [24,25]. Moreover, data from other studies showed that EFR3A and TTC7 homologs (TTC7A and TTC7B) interact with FAM126A (hyccin) [29], a protein of previously unknown function. FAM126A-deficient fibroblasts obtained from patients with hypomyelination and congenital cataract (HCC) exhibited significant defects in the PI4KA complex formation and a decrease in PI4P levels. Interestingly, the HCC patient fibroblast cells devoid of FAM126A showed increased levels of the FAM126B paralog, which could partially compensate for the lack of FAM126A [30].

Performed in 2016, a proximity biotinylation-based MS/MS study (BioID) on a human osteosarcoma cell line demonstrated that EFR3A is a binding partner of kindlin-2, a component of the focal adhesion complexes that interacts with phosphoinositides and integrins at the plasma membrane [31]. The interaction between EFR3A and kindlin-2 has been proposed to control the PIP2 concentration and regulate the local endocytosis [32]. Another BioID experiment identified EFR3A as a top interactor of NRAS, HRAS, and KRAS oncoproteins. EFR3A and its paralog EFR3B were found to play an essential role in recruiting mutant RAS to the plasma membrane and organizing it into “functional, oncogenic” nanoclusters. Down-regulation of EFR3A in HEK-HT cells (cell line with the prevalent activating mutation in *KRAS* oncogene, *KRAS^G12C^*) prevented KRAS^G12V^ accumulation at the PM and reduced the oncogenic signaling via reduced levels of downstream signaling molecules p-ERK and p-AKT [33].

Further studies [34] revealed that the EFR3s-PI4KA complex facilitates the non-random distribution of glucose transporter 4 (GLUT4) within the plasma membrane and controls insulin-stimulated glucose transport. EFR3s and PI4KA showed elevated expression during differentiation of murine adipocytes (3T3-L1 cell line) and accumulated at the plasma membrane in response to insulin. Knock-down of either *EFR3A* (the experiment did not include *EFR3B*) or PI4KA disturbed insulin-stimulated glucose transport in 3T3-L1 cells [34,35].

Studies on brain-specific deletion of *EFR3A* indicate its role in hippocampal neurogenesis by maintaining survival and maturation of newborn neurons and decreasing the number of apoptotic cells in the hippocampus of EFR3A-deficient mice due to increased levels of the BDNF (brain-derived neurotrophic factor)-TrkB (tropomyosin-related kinase B) signaling pathway molecules [36]. Advanced techniques such as cryo-EM and HDX-MS, including mutational analysis, revealed the structural basis of EFR3A and the PI4KA complex interaction and, consequently, PI4KA regulation at the plasma membrane [3].

**Figure 2:**
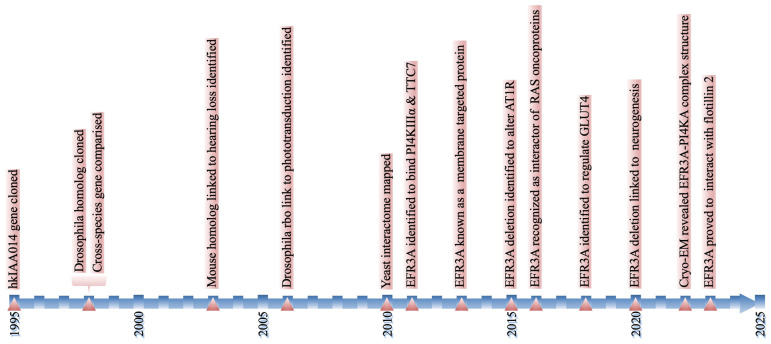
Thirty years of EFR3 research. A simplified representation of the EFR3A discovery history. For details, see the text above.

Our recent study [37] identified flotillin 2 (FLOT2) as a potential binding partner of human EFR3A. The interaction between the proteins was discovered in a pull-down experiment (using FLOT2 as the bait) and verified using a far Western blot. The study showed that the down-regulation of EFR3A impairs the lateral organization of the plasma membrane and influences epidermal growth factor receptor (EGFR) and phospholipase C gamma phosphorylation [37]. A simplified representation of the EFR3A discovery history is shown in Figure 2.

The literature on human EFR3A, regarding its various biological functions and interactions, has so far provided only basic information about the nature of the protein. Previous research has largely focused on either EFR3 paralog individually, with limited attention to the fact that these proteins are co-expressed and may exhibit functional redundancy. In this short review, we focus specifically on the possible role of EFR3A in human disease states, as the literature on this topic is significantly more advanced than that of EFR3B.

## 3. Mutations and Changes in *EFR3A* Expression Levels Are Linked to Human Pathologies

### 3.1. Neurological Disorders

The very first link between EFR3A and pathological states in humans was reported for autism spectrum disorders (ASDs) in 2014. ASDs occur in about 1 in 100 children. ASD encompasses a spectrum of neurodevelopmental disabilities, defined by persistent deficits in social communication and social interaction and restricted repetitive patterns of behavior, interests, or activities. Children with ASD have intellectual disabilities, language problems, and even epilepsy (for review see, e.g., [38]). Currently, the causes of ASD are not known but it is assumed that idiopathic ASD is associated with inherited mutations and environmental condition influence. Gupta et al. [39] showed that rare (<1%), somatic, non-synonymous mutations altering the amino acid sequence of EFR3A and potentially affecting its structure were found to occur 2.08 times more frequently (0.73% vs. 0.35%) in autistic patients compared to control subjects. The correlation between *EFR3A* and ASD was strengthened by the fact that it shared the expression pattern (*p* < 2.2 × 10^−16^) with ASD significantly associated genes [40] (synaptic genes and PI(4,5)P_2_ phosphatase) both in the fetal and mature human brain and was proven to be involved in synaptic phosphoinositide metabolism. The six different deleterious mutations in *EFR3A* identified in the autistic patients’ cohort were recognized and extensively validated in a large whole-exome and a Sanger sequencing study involving 2196 cases and 3389 controls. Although the results suggested that the mutations in *EFR3A* may be associated with an increased risk of developing ASD and contribute to its genetic heterogeneity, their low frequency questioned their value as a diagnostic tool [39]. Further research is needed to understand the role of *EFR3A* in the development of ASD, and to clarify whether additional mechanisms involving *EFR3A* are associated with the spectrum of TRP phenotypes.

Another link between *EFR3A* and neurological diseases was unveiled by Zhao et al., [41] who identified *EFR3A* among fourteen hub genes predictive of seizures in patients diagnosed with primary glioma (area under the ROC curve for the signature: 0.9). The 14-gene classifier successfully stratified subjects into low- and high-risk groups, with a significance level of *p* < 0.001. The hazard ratio for *EFR3A* alone was 1.47, while the risk ratios for all the genes in the study ranged from 0.15 to 5.55 [41]. A better understanding of the *EFR3A* gene in epilepsy requires further studies, but taking into account that the pathogenesis of epilepsy is associated with calcium-related pathways and synaptic signaling, the *EFR3A* gene could be a good candidate for further studies. A recent study by Gao et al. [42] identified an association between EFR3A and essential tremor (ET), the most common neurological disorder affecting all age groups (prevalence 0.4–0.9%), which manifests in involuntary shaking. Due to the overlap of symptoms between ET and other neurological disorders, such as Parkinson’s disease, the diagnosis of ET remains challenging. There is an urgent need to develop novel and accurate biomarkers to facilitate accurate diagnosis of this disease. Analysis of public (GSE134878) and original RNA-sequencing profiles obtained for peripheral blood mononuclear cells (PBMCs) of 23 healthy controls and 35 ET patients revealed abnormalities in the expression pattern of *EFR3A* and six other genes. Although *EFR3A* was significantly up-regulated in ET patients in both RNA-seq datasets (no statistics provided), the gene was not included in further validation [42].

Other studies indicate a potential role of *EFR3A* in the development of Alzheimer’s disease. Conditional knockout of *EFR3A* in the CA3 or CA1 region of the hippocampus in mice inhibits amyloid Aβ-induced presynaptic PIP2 hydrolysis and aberrant neurotransmitter release at the synapse between the Schaffer collateral (SC) and CA1 pyramidal neurons, which ultimately leads to restoration cognitive function and memory in APP/PS1 mice (model for Alzheimer’s disease) [43]. In contrast, the depletion of the highly abundant *EFR3B* isoform in the CA2/CA3 regions of pyramidal neurons (PN) led to impaired excitability and deficits in the recognition of social novelty in mice [44].

Research on EFR3A in human neurological conditions is still in its early stages. There is no available data regarding the “behavior” of the EFR3B paralog in the EFR3A-malfunction background. In our opinion, to better understand the molecular mechanisms underlying neurological diseases, it is also important to analyze the functionality of both paralogs in a given group of diseases.

### 3.2. Cardiovascular Diseases

Coronary artery disease (CAD) is the leading cause of death globally, resulting from chronic vascular inflammation and endothelial dysfunction [45]. There is a deficit of non-invasive biomarkers with the potential to detect early-stage CAD and predict disease complications. The study by Sun and collaborators [46] revealed increased levels of *EFR3A* mRNA in the plasma of 60 patients diagnosed with coronary artery disease. *EFR3A* was found to be one of the targets of miR-367, a known negative regulator of inflammatory responses [47]. Increased levels of *EFR3A* transcript in the plasma of CAD patients correlated with diminished amounts of miR-367 and over-activation of NF-κB inflammatory cascade in plasma samples. The impact of miR-367 on *EFR3A* expression was also noticeable in the human aortic endothelial cell line (HAEC) transfected with miR-367 mimic. HAECs with greater expression of this miRNA exhibited a decreased expression of *EFR3A* and lower activity of the NF-κB pathway compared to unaffected cells. Given that miR-367 is dysregulated in numerous pathologies associated with chronic inflammation, including cancer, dementia, stroke, and diabetes, the expression of *EFR3A* may also be susceptible to changes in these conditions [46]. It should be noted that the latest research concerning Lyme borreliosis indicated that the EFR3A protein is engaged in the regulation of IL-1β responses in PBMCs (one of the strongest associations with IL-1β responses upon Bb stimulation of these cells) [48]. This may support the participation of EFR3A in the regulation of inflammatory response.

Another study pointed out a strong association between a single nucleotide polymorphism (SNP) in the *EFR3A* loci (rs4736529) and major adverse cardiovascular events (MACEs) in patients with acute coronary syndromes (ACSs) [49] subjected to antiplatelet therapy (after artery opening). An ACS is a type of CAD manifesting as unstable angina, frequently leading to myocardial infarction (heart attack), coronary revascularization, stroke, heart failure, and death (MACEs) [49]. To lower the risk of MACE, patients undergo dual therapy with clopidogrel and aspirin (after coronary angioplasty), which is not always effective. The study by Liu et al. [50] identified a set of SNPs (including an *EFR3A* variant) that showed a great performance in predicting 18-month MACEs (AUC 0.92–0.94) in ACS patients subjected to pharmacological treatment. Significant variants were identified via whole exome sequencing performed on 51 samples of patients experiencing major adverse cardiovascular events (MACEs) and 117 samples obtained from subjects with no clinical events. Downstream targeted validation performed on a group of 1793 ACS patients (123 MACE, 1580 no-MACE patients) confirmed the predictive power of the SNP signature. The *EFR3A* variant was associated with an increased risk of MACE with an odds ratio of 3.16 and a 95 percent confidence of 1.04–9.61. The mentioned study did not verify whether rs4736529 affects the expression of *EFR3A*. External validation is essential to establish the reproducibility of the SNP classifier in predicting MACEs and to assess its potential as a clinical tool [50].

It should be noted that currently, no reports are linking the *EFR3B* paralog to human cardiovascular pathologies. It would be reasonable to investigate the mutation and expression landscape of both EFR3s in this group of diseases.

### 3.3. Colorectal Cancer

The first mention of the possible link between *EFR3A* and cancer appeared in the work by Zhou [51], who investigated somatic genetic changes accumulating during the progression of colorectal adenoma to colorectal carcinoma (CRC). Whole-exome sequencing performed on matched normal mucosa, adenoma (pre-malignant lesion), and adenocarcinoma tissue from the same patient revealed 12 non-synonymous somatic mutations in the adenoma and 42 nonsense variants in the CRC. None of the mutations were common between the pre-cancerous and cancerous stages. The signature of adenoma included a novel missense variant in the *EFR3A* gene (chr8: 133057465; p.G390E), which was not further found in the validation cohort of 288 CRC cases (non-recurrent mutation) [51]. It is unclear whether the *EFR3A* variant represented a rare, non-significant mutation event in the adenoma or if it was overlooked by Sanger sequencing due to its low frequency. It should be noted that the study of others on the whole-exome sequencing of colorectal adenoma did not mention mutation(s) in this gene [52].

Another putative relationship between *EFR3A* and colorectal cancer has been described by Li and Han [53]. The authors evidenced that the PI4KA-TTC7-FAM126-EFR3 complex is heavily guarded in the CRC against significant genetic perturbations in any of its components. In many of the CRC cell lines, the expression of FAM126B, TTC7B, and EFR3B paralogs is low, making these cells dependent on EFR3A, FAM126A, or TTC7A. In other words, in normal cells, the loss of a single paralog did not inhibit the assembly of the lipid kinase complex and downstream signaling because the second unaffected paralog could compensate for the deficiency. The phenomenon, known as synthetic lethality (SL), constitutes a protection mechanism that allows the cells to survive in an environment deprived of the crucial molecular regulator, e.g., major protein paralog via substituting it with its paralog protein [53,54,55].

CRC is the third most common malignant neoplasm in terms of incidence and the second most common cause of cancer-related deaths worldwide (statistics from 2022) [56]. At present, no effective molecular tools exist that would facilitate early detection of CRC, predicting its course and response to treatment. Elucidating the exact architecture, and the plasticity of the PI4KA complex, as well as the PI4KIA-associated SL phenomenon, is essential for gaining a deeper understanding of CRC and developing novel, diagnostic, and therapeutic options.

### 3.4. Pancreatic Ductal Adenocarcinoma

Pancreatic ductal adenocarcinoma (PDAC) is the most common and most aggressive histologic type of pancreatic cancer, which ranks third in worldwide cancer death statistics. The malignancy is considered the most KRAS-dependent of all cancers, as more than 85% of PDAC cases carry activating mutations in the *KRAS* gene (reviews, e.g., [56,57,58]). The majority of hotspot mutations in *KRAS* appear in the 12 codons and include G12D (39.2%), G12V (32.5%), G12R (17.1%), Q61H/R/K (6.5%), G12C (1.7%), G12S/L/I (1.4%), G13D/P/H/R (1.4%), and others (0.5%) [58]. KRAS oncoprotein is a highly challenging, almost undruggable target due to the resistance acquired during therapy. Targeting other components of the KRAS interactome may comprise an avenue to overcome the resistance mechanism. A recent BioID study identified EFR3A [33] as a member of the RAS interactome and a direct binding partner of the oncogenic (but not wild-type) form. EFR3A was found to impact the RAS signaling by recruiting overactive KRAS to the PM, facilitating its organization into functional nanoclusters composed of 6–7 RAS proteins. Analysis of the spectrum of genetic alterations in *EFR3A* across all human cancers (cBioportal cancer genomics datasets, *n* = 10,967 samples in total) revealed that PDAC exhibits the highest *EFR3A* amplification (almost 12% of cases) among all neoplasms. Patients with *EFR3A* gene mutations presented ~26% lower overall median survival and ~53% reduction in disease-free progression compared to subjects with normal *EFR3A* (15.3 vs. 20.8 months; 9.6 vs. 20.5 months). The expression level of *EFR3A* was significantly raised in the PDAC cohort, especially in *KRAS* mutation-positive subjects (179 PDAC vs. 171 normal cases, *p* < 0.0001). Disruption of *EFR3A* in HEK-HT kidney embryonic cells harboring KRAS^G12V^ resulted in a drastic decrease in PI4P, phosphatidylserine, and KRAS levels at the PM and reduced RAS signaling [33]. Targeting the PI4KA pathway in KRAS mutation-positive PDAC appears a highly promising direction toward pancreatic cancer treatment.

### 3.5. Nasopharyngeal Cancer

Nasopharyngeal carcinoma (NPC) is a rare type of cancer (1 case per 100,000 in Europe) endemic to regions such as Asia, North Africa, the Middle East, and Alaska (25–50 cases per 100,000) [59]. In the majority of patients (~70%), the disease progresses to an advanced stage, which is associated with an unfavorable prognosis. Understanding the mechanism behind NPC and identifying novel therapeutic targets remain the highest priority in current NPC research. In 2020, a high-throughput circular RNA sequencing study identified circEFR3A (hsa_circ_0135761) among the top dysregulated circRNAs in NPC (log2 Fold Change = 5.39, *p* = 0.00316). The circEFR3A expression was observed in four, matched NPC-normal tissue samples [60]. Three years later, Jiang and Xia [61] confirmed the overexpression of circEFR3A in human NPC cell lines (C666-1, SUNE1, 5-8F and 6-10B, NP69), and found that circEFR3A promotes the expression of *EFR3A*. The silencing of circular RNA EFR3A (circEFR3A) had an anti-oncogenic effect, as it resulted in increased apoptosis and decreased cell proliferation [61]. The positive interplay between the oncogenic circEFR3A and *EFR3A* expression seems an attractive target for novel diagnostic and therapeutic options for NPC. However, we need to keep in mind all the limitations connected with circRNA’s research recently reviewed in [62].

### 3.6. Brain Tumors

Adult and pediatric brain tumors represent a diverse set of diseases that pose significant challenges in early diagnosis and treatment (see reviews, e.g., [63,64]). Identifying effective drug targets for brain cancers is particularly difficult because tumors with the same histological features often exhibit little or no overlap in their mutational, epigenetic, and chromosomal rearrangement profiles. Functional genetic lethal screens and computational modeling were used to uncover common and unique genetic dependencies in adult and pediatric brain tumors. The study [65] utilized the data from the authors’ own CRISPR-Cas9 lethality screens performed on single adult glioma and five pediatric brain tumors (gliomas, medulloblastoma, and teratoid rhabdoid tumors) and took advantage of the gene dependency networks obtained earlier for >900 human cancer cell lines [66]. The high-throughput analysis identified a strong genetic dependency between *EFR3A* and *EFR3B*, both in adult and children’s brain cancer tissues [65]. In vitro, validation experiments performed on glioblastoma cell line GSC-0827 expressing high levels of *EFR3B* (*EFR3B^high^*) and glioblastoma GSC-0131 cell line with trace expression of *EFR3B* (*EFR3B^low^*) confirmed the compensatory relationship between *EFR3A* and *EFR3B*. Depletion of *EFR3A* in *EFR3B^low^* cells resulted in a striking (50%) reduction in cellular viability, whereas its elimination from *EFR3B^hig^*^h^ cells had no effect. Moreover, ectopic expression of *EFR3B* in *EFR3B^low^* cells restored cell viability to normal levels [65]. Cited results may again point to the role of paralog genes in cancer biology and future therapies.

## 4. Conclusions

The *EFR3A* gene and protein are critical yet underexplored elements in the complex landscape of human diseases. This review aims to summarize the diverse roles of EFR3A in various human pathologies, including neurological disorders, cardiovascular diseases, and cancer (Table 1). Further basic research is needed to clarify the potential clinical applications of *EFR3A*/EFR3A, whether as a biomarker for certain diseases or as a therapeutic target, particularly in gene therapy for specific neoplasms. An important consideration in these studies is the variety of isoforms, especially the existence of paralogs that may potentially compensate for the major isoform.

## Figures and Tables

**Table 1:** EFR3A in human disease states.

Disease Category	Associated Findings	References
Neurological Disorders	EFR3A mutations linked to autism spectrum disorder (ASD).	[39]
	*EFR3A* identified as a hub gene predicting seizures in primary glioma patients.	[41]
	Association with essential tremor.	[42]
	Reducing *EFR3A* gene expression prevents the SC-CA1 synapse dysfunction and rescues synaptic and spatial learning and memory deficits in Alzheimer’s disease mouse model.	[43]
	Depletion of the *EFR3B* isoform in the CA2/CA3 regions of pyramidal neurons (PNs) led to impaired excitability and deficits in the recognition of social novelty in mice.	[44]
Cardiovascular Diseases	Overexpression of *EFR3A* linked to coronary artery disease (CAD), regulated by miR-367 and affecting inflammatory pathways.	[46]
	SNP in EFR3A associated with major adverse cardiovascular events (MACEs) in acute coronary syndrome patients.	[50]
Oncological Diseases	Colorectal Cancer (CRC):	
	Somatic mutation identified in adenoma during adenoma-to-carcinoma progression.	[51,52]
	Protective synthetic lethality mechanism involving EFR3A in CRC.	[53,54,55]
	Pancreatic Ductal Adenocarcinoma (PDAC):	
	EFR3A identified as a player in KRAS-mutant PDAC, affecting survival and tumor progression.	[33]
	Nasopharyngeal Carcinoma (NPC):	
	circEFR3A overexpression promotes NPC growth; silencing circEFR3A shows anti-oncogenic effects.	[60,61]
	Brain Tumors:	
	Strong genetic dependency between EFR3A and EFR3B, affecting cell viability in glioblastoma cell lines.	[65]

## Data Availability

Not applicable.

## Abbreviations

The following abbreviations are used in this manuscript:

ACS	acute coronary syndrome
ASD	autism spectrum disorder
AT1R	angiotensin II receptor type 1
BDNF	brain-derived neurotrophic factor
CAD	coronary artery disease
CRC	colorectal cancer
EFR3A	eighty five requiring 3
ET	essential tremor
HCC	hypomyelination and congenital cataract
HDX-MS	hydrogen deuterium exchange mass spectrometry
MACE	major adverse cardiovascular events
NPC	nasopharyngeal carcinoma
PDAC	pancreatic ductal adenocarcinoma
PI(4,5)P_2_	phosphatidyl inositol 4,5 bisphosphate
PI4KA	phosphatidylinositol 4-kinase alpha
PLC	phospholipase C
PM	plasma membrane
SNP	single nucleotide polymorphism
TRP	transient receptor potential
TTC7	tetratricopeptide repeat domain 7
